# Supplementary material for: Formin-like 1 (FMNL1) Is Associated with Glioblastoma Multiforme Mesenchymal Subtype and Independently Predicts Poor Prognosis
Source: Int J Mol Sci. 2019 Dec 17;20(24):6355. doi: 10.3390/ijms20246355 (PMC6940780; doi:10.3390/ijms20246355)
Supplement: Supplementary file 1 [file ijms-20-06355-s001.zip › supplementary figure for IJMS1128v2.pdf]

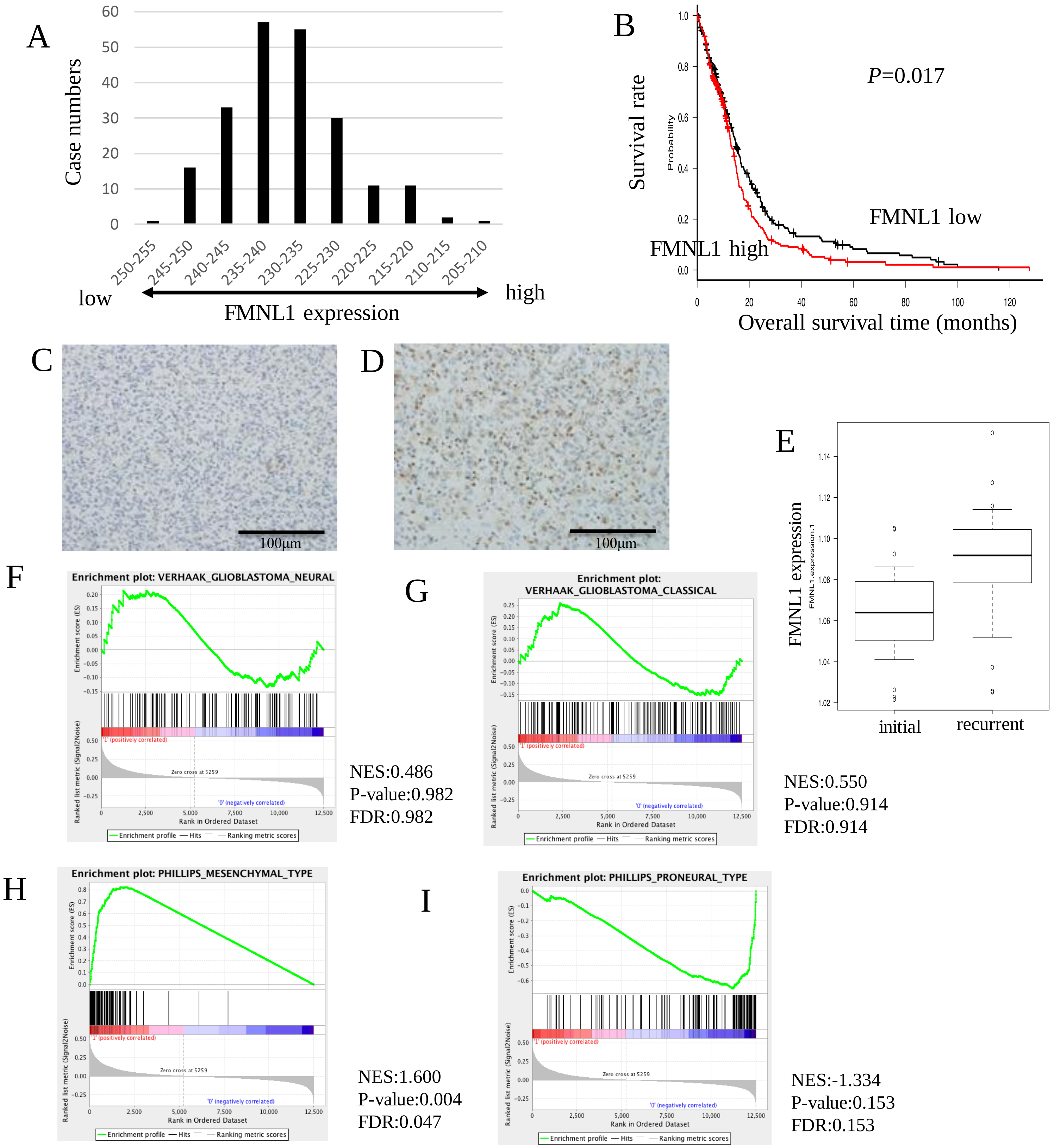

Supplementary Figure S1.

A, The distribution plot of FMNL1 immunohistochemical expression levels. **B,** Kaplan-Meier analysis of overall survival in TCGA GBM patients high or low expressing FMNL1. The former exhibited significantly poorer prognoses than the latter (P = 0.017). **C-E,** Representative immunohistochemical staining for FMNL1 in a primary (C) and recurrent tumour (D) are shown, and were compared by paired t-test (P < 0.01, E). **F-I,** Enrichment plot of FMNL1 mRNA expression in GBM specimens from TCGA for the gene sets VERHAAK_GLIOBLASTOMA_CLASSICAL,VERHAAK_GLIOBLASTOMA_NEURAL, PHILLIPS_GLIOBLASTOMA_MESENCHYMAL, and PHILLIPS_GLIOBLASTOMA_PRONEURAL. NES, normalized enrichment score; P, nominal P-value; FDR, False discovery rate.

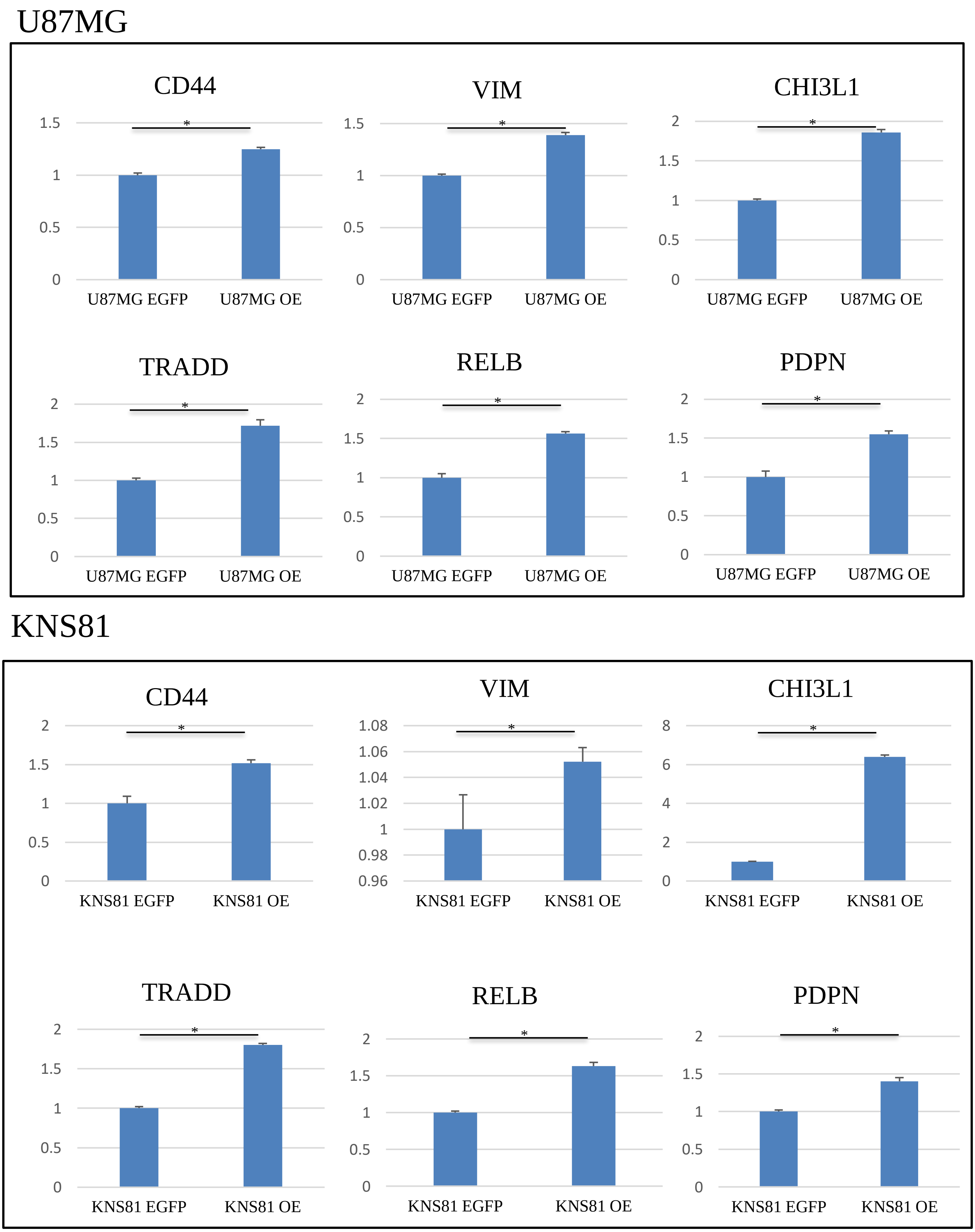

Supplementary Figure S2.

FMNL1 induces mesenchymal markers expression. Mesenchymal markers, measured by RT-PCR, were significantly upregulated in U87MG and KNS81 cells overexpressing FMNL1, compared with cells overexpressing EGFP. *$P < 0.01$

U251MG

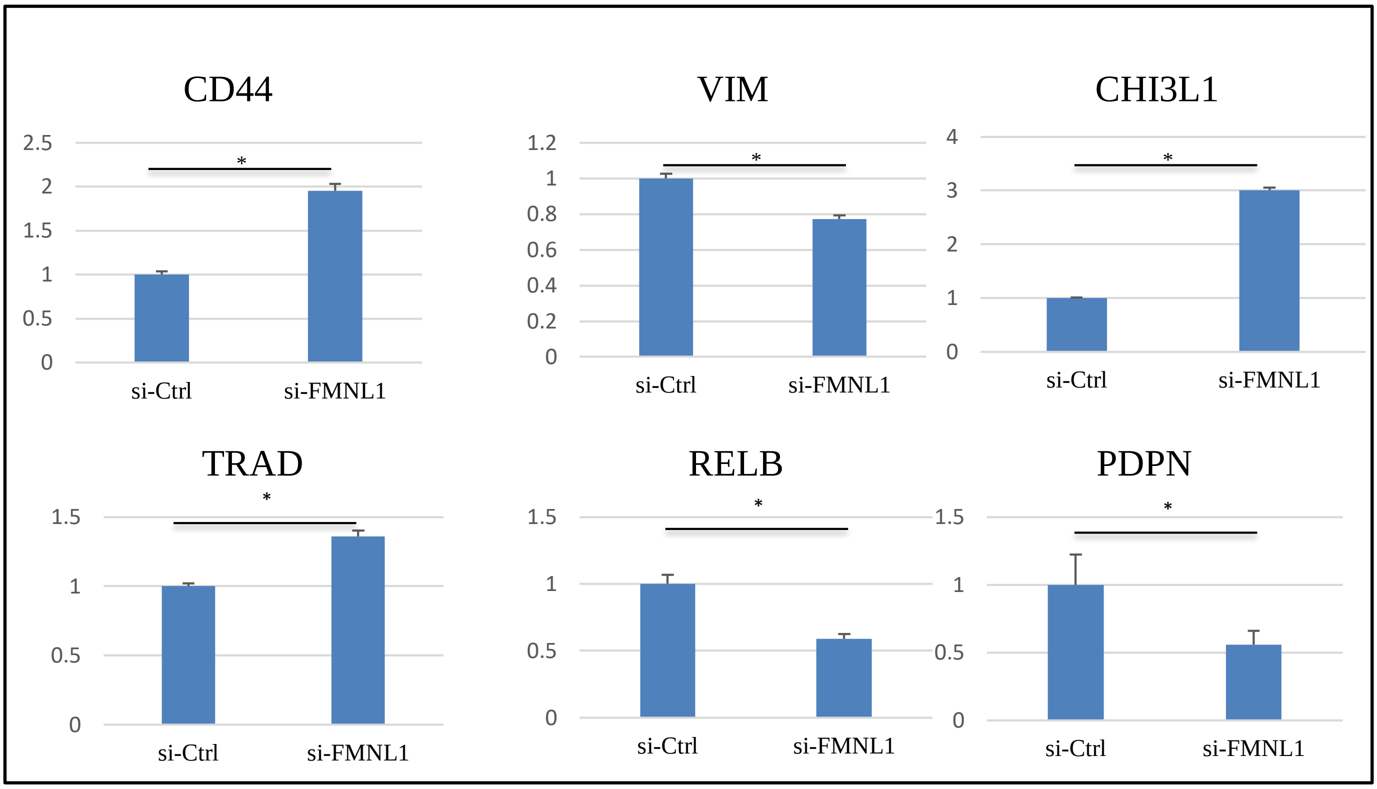

DBTR-05MG

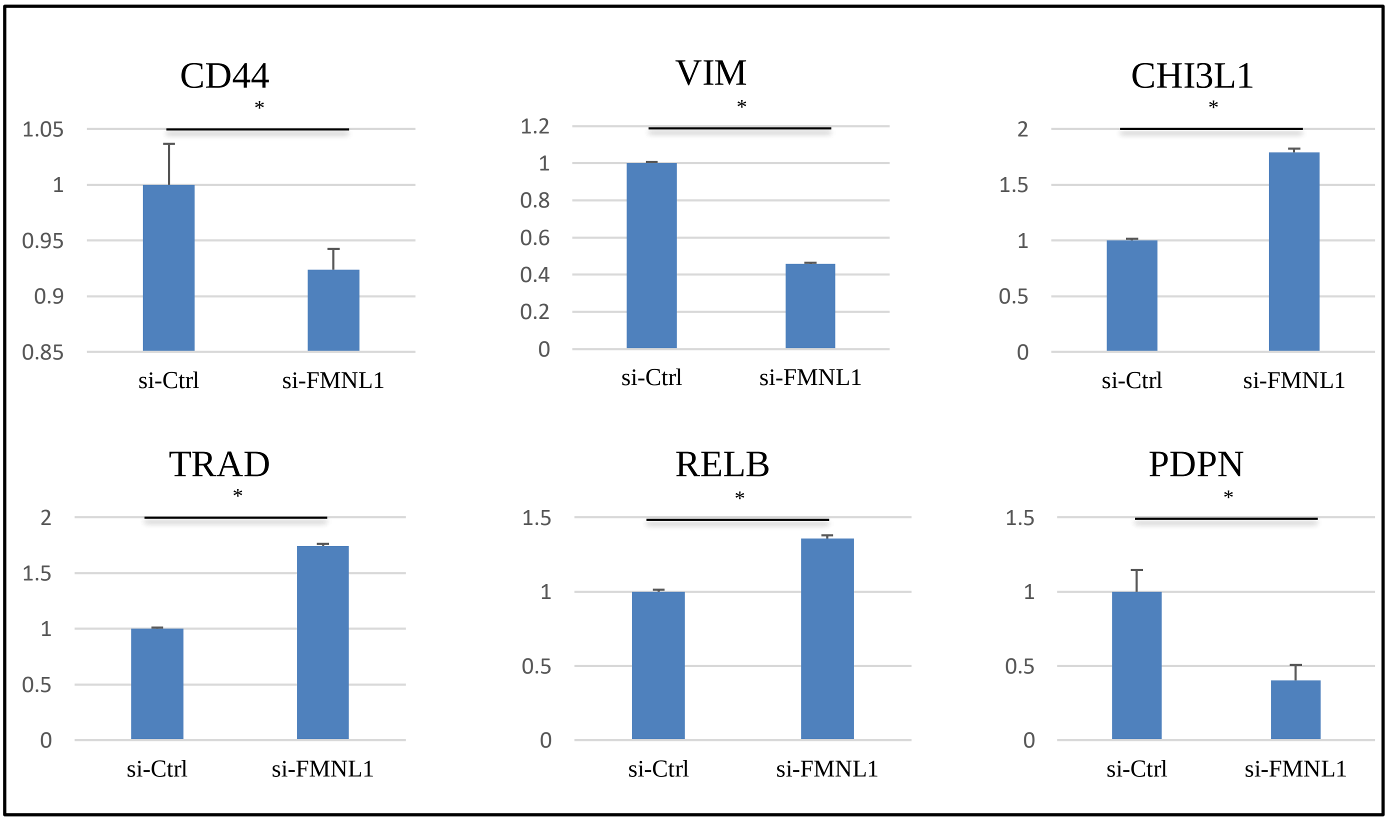

Supplementary Figure S3.

FMNL1 knockdown effect on mesenchymal markers. *$P < 0.01$

U251MG

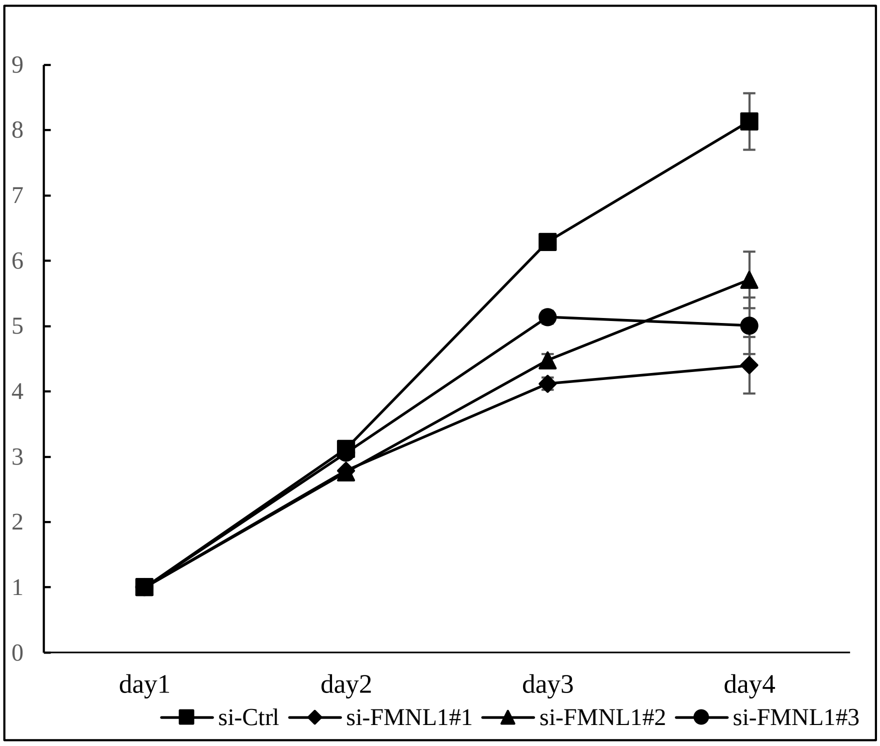

DBTRG-05MG

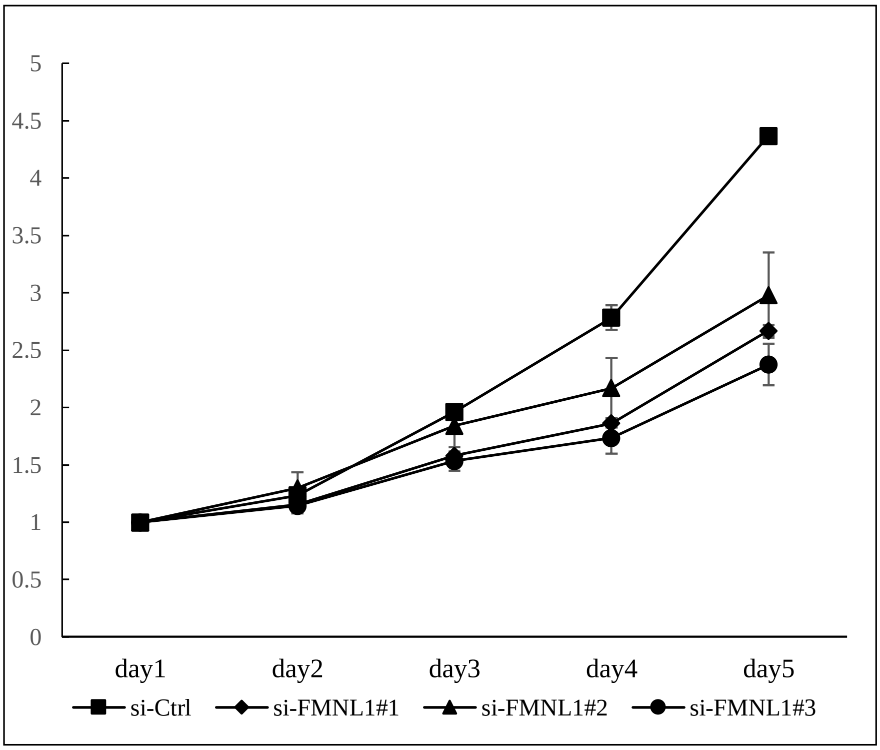

Supplementary Figure S4.

FMNL1 knockdown effect on GBM cell proliferation.
Cell viability was measured using the MTT assay.

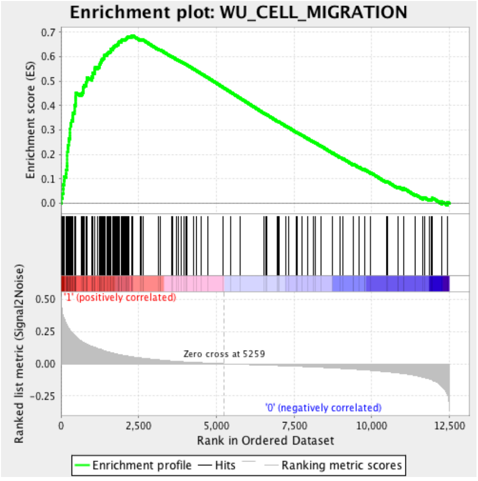

NES:1.616
P-value:0.0
FDR:0.054

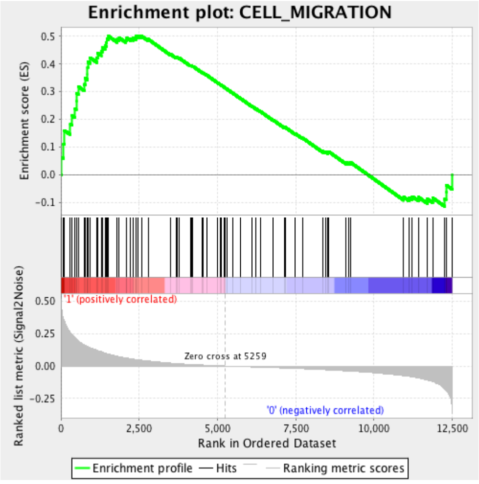

NES:1.522
P-value:0.014
FDR:0.053

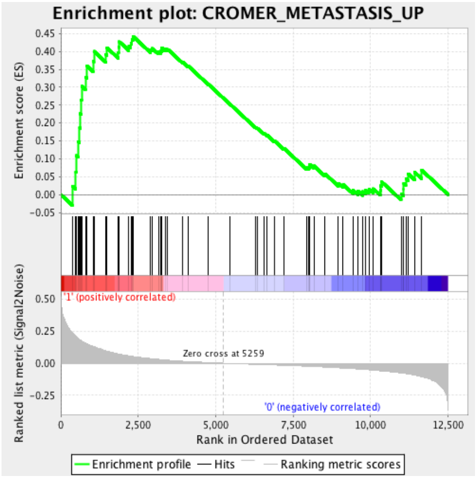

NES:1.623
P-
value:0.014
FDR:[illegible]

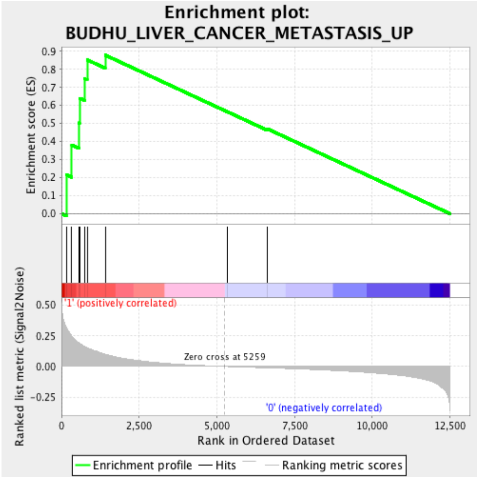

NES:1.553
P-value:0.004
FDR:0.052

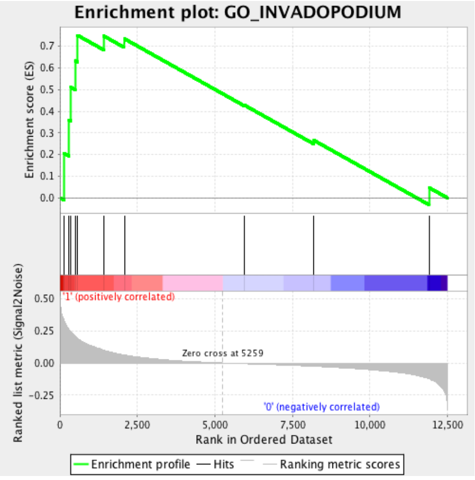

NES:1.388
P-value:0.068
FDR:0.108

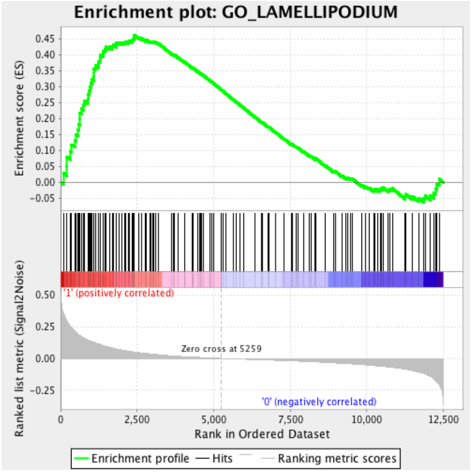

NES:1.600
P-value:0.011
FDR:0.053

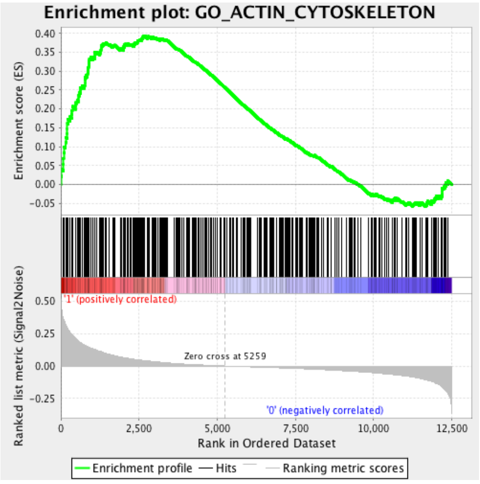

NES:1.556
P-value:0.002
FDR:0.055

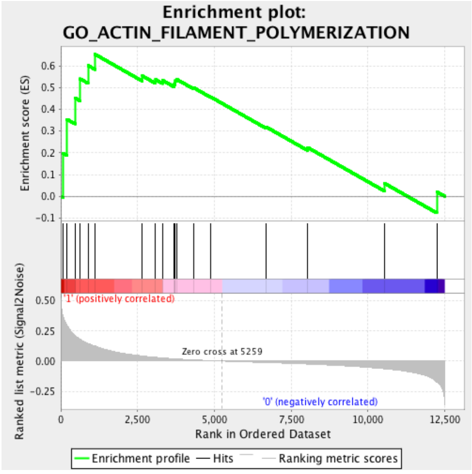

NES:1.682
P-value:0.015
FDR:0.015

Supplementary Figure S5.

Enrichment plot of FMNL1 mRNA expression in TCGA GBM patients for the gene sets GO_LAMELLIPODIUM, KEGG_FOCAL_ADHESION, GO_INVADOPODIUM, BUDHU_LIVER_CANCER_METASTASIS_UP, CROMER_METASTASIS_UP, CELL_MIGRATION, WU_CELL_MIGRATION, GO_ACTIN_CYTOSKELETON, and GO_ACTIN_FILAMENT_POLYMERIZATION. NES, normalized enrichment score; P, nominal *P*-value; FDR, false discovery rate.
