# Supplementary material for: Formin-like 1 (FMNL1) Is Associated with Glioblastoma Multiforme Mesenchymal Subtype and Independently Predicts Poor Prognosis
Source: Int J Mol Sci. 2019 Dec 17;20(24):6355. doi: 10.3390/ijms20246355 (PMC6940780; doi:10.3390/ijms20246355)
Supplement: Supplementary file 1 [file ijms-20-06355-s001.zip › supplementary table for IJMSpdf.pdf]

Relationship between FMNL1 expression and clinical GBM features

		Total n=217	%	FMNL1 expression				P-value
				High n=109	%	Low n=108	%	
Age(mean±SD)		64.92±15.12		67.7±13.93		62.1±15.87		0.006
Gender								
	Male	122	56.2	66	60.6	56	51.9	0.219
	Female	95	43.8	43	39.4	52	48.1	
Location								
	Dominant side	98	45.2	53	48.6	45	41.7	0.129
	Non-dominant side	89	41.0	37	33.9	52	48.1	
	Bilateral	27	12.4	17	15.6	10	9.30	
	Posterior fossa	3	1.40	2	1.80	1	0.90	
Karnofsky performance status								
	0-70	128	59.0	72	66.1	56	51.9	0.039
	80-100	89	41.0	37	33.9	52	48.1	
Extent of surgical resection								
	Total+subtotal removal	119	54.8	50	45.9	69	63.9	0.010
	Partial removal+biopsy	98	45.2	59	54.1	39	36.1	
Number of surgeries								
	Single	185	85.3	104	95.4	81	75.0	<0.001
	Multiple	32	14.7	5	4.60	27	25.0	

Groups were compared by $\chi 2$ test with $P < 0.05$ considered statistically significant.

Supplementary Table S1

Gene	Forward sequence	Reverse sequence
**GAPDH**	TGCACCACCAACTGCTTAG	GAGGCAGGGATGATGTTC
**FMNL1**	GGTCCTCCTGATGCCCTAGGAA	TGTGAAGACAGTGCCGGTGA
**CHI3L1**	CTCAAGAACAGGAACCCCAA	TCTGGGTGTTGGAGGCTATC
**CD44**	CTGATCATCTTGGCATCCCT	AGCTTTTTCTTCTGCCCACA
**VIM**	TGCCCTTAAAGGAACCAATG	GCTTCAACGGCAAAGTTCTC
**RELB**	CAGCCTCGTGGGGAAAGAC	GCCCAGGTTGTTAAAACTGTGC
**TRADD**	GCTGTTTGAGTTGCATCCTAGC	CCGCACTTCAGATTTCGCA
**PDPN**	ACCAGTCACTCCACGGAGAAA	GGTCACTGTTGACAAACCATCT

Supplementary Table S2
